# A Smart Chatbot for Interactive Management in Beta Thalassemia Patients

**DOI:** 10.1155/2022/9734518

**Published:** 2022-05-11

**Authors:** Alma Mohammed Alturaiki, Haneen Reda Banjar, Ahmed Salleh Barefah, Salwa Abdulrahman Alnajjar, Salwa Hindawi

**Affiliations:** ^1^King Abdulaziz and His Companions Foundation for Giftedness and Creativity (Mawhiba), Saudi Arabia; ^2^Computer Science Department, Faculty of Computing and Information Technology, King Abdulaziz University, Jeddah, Saudi Arabia; ^3^Centre of Artificial intelligence in Precision Medicines, King Abdulaziz University, Jeddah, Saudi Arabia; ^4^Hematology Department, Faculty of Medicine, King Abdulaziz University, Jeddah, Saudi Arabia; ^5^Hematology Research Unit, King Fahd Medical Research Centre, King Abdulaziz University, Jeddah, Saudi Arabia; ^6^Saudi Friends of Thalassemia and Sickle Cell Anaemia Society, Jeddah, Saudi Arabia

## Abstract

**Background:**

*β*-thalassemia is an inherited blood disorder that affects the production of hemoglobin molecules owing to the reduction or absence of beta chains. Transfusion therapy has had a key role in extending the lifespan of *β*-thalassemia patients. This life-saving therapy is linked to numerous assessments and complications that now comprise most thalassemia management considerations. Consequently, many patients do not receive adequate information about the required assessments, as indicated by evidence-based medical guidelines. Patients with *β*-thalassemia may benefit from chatbots that follow up on their condition and that provide the required assessment information. Self-management will hopefully have a positive impact on health outcomes.

**Objectives:**

This study aims to develop a chatbot that can assist in the management of *β*-thalassemia by providing the assessment information required to monitor patients' statuses.

**Methods:**

The chatbot operated as a messaging system. A question/answer system was created based on knowledge pertaining to *β*-thalassemia assembled from experts, medical guidelines, and articles. Recommendations regarding the patient's follow-up assessment are made based on the answers.

**Results:**

A prototype was implemented to demonstrate how the chatbots could dynamically and flexibly provide the assessment information required to follow up on and monitor patients. A small sample of adults with *β*-thalassemia used the chatbot to examine the system's usability and perceived utility. A system usability scale and utility scale were implemented to complete a post-test survey. The chatbots were considered by 34 patients, of whom the majority (72%) found them easy to use, while more than 90% of patients considered their use beneficial. Most of the participants agreed that the chatbots could improve their knowledge about their *β*-thalassemia assessments.

**Conclusion:**

Our findings suggest that chatbots can be beneficial to the development of recommended tests and management related to the assessment of *β*-thalassemia.

## 1. Introduction

*β*-thalassemia is one of the two most common inherited blood disorders that affect the production of hemoglobin molecules. Hemoglobin molecules are composed of two beta chains and two alpha chains; the reduced synthesis (*β*^+^) or absence (*β*^o^) of the chain *β*-globin in the HbA molecule is characterized by *β*-thalassemia [1]. Recent statistics reveal that 7% of the world's population has been diagnosed with hemoglobin disorders, and more than 50,000 children die from thalassemia major every year [2]. Hemoglobin disorders are worldwide compared with other monogenic disorders [3].

Transfusion therapy has had a key role in extending the lifespan of a patient with *β*-thalassemia. This life-saving therapy is linked to numerous assessments and complications, which now comprise most thalassemia management considerations. Consequently, many patients do not receive adequate information about the required assessments, as outlined by evidence-based guidelines. Moreover, healthcare systems that are efficient must have accessibility and availability [4]. Thus, a patient with *β*-thalassemia may benefit from chatbots that follow up on their condition and provide the requisite assessment information. A chatbot is a software system with an interactive interface that can be utilized by patients or physicians to provide personalized, real-time feedback and to complete various tasks with the aim of obtaining knowledge [5]. Chatbots function as virtual conversational agents that imitate human interactions and could provide direct and cost-effective medical advice to patients to increase their knowledge about the required assessments and tests that will hopefully have a positive impact on their health outcomes.

Rule-based systems have been involved in decision support, management, and accurate diagnosis of *β*-thalassemia. Hasseini Eshphala et al. [6] reported the use of an artificial neutral network, which is a computational model that uses complex calculation to diagnose patients with *β*-thalassemia and iron deficiency anemia. Banjar et al. [7] also designed a web-based expert system to manage *β*-thalassemia, focusing on treatment recommendations. Their approach has gained attention [8–10], and experiments have been performed on not only *β*-thalassemia but also other disorders and diseases using rule-based expert systems that have yielded positive results. As a web-based expert system [7], chatbots offer distinct advantages when responding to well-defined questions, thus providing a convenient and reliable approach to implementing question/answer systems in knowledge-focused fields, such as the medical field. Xiangmin et al. [4] aimed to create a chatbot that would allow patients of any disease to self-diagnose. The authors applied a data-driven method to investigate the system log of DoctorBot collected between September 2018 and March 2019 to better understand how self-diagnosis chatbots are employed in the real world. During six months, 16,519 users initiated 47,684 consultation sessions. A session identification (ID), the user's general information, consultation details, and a diagnostic report automatically generated by the chatbot once the consultation was concluded, and user input were included in each consultation session's log. All these studies have involved rule-based expert systems in diagnosing or managing disorders and diseases. One study utilized a web-based expert system to manage *β*-thalassemia [7], a second study employed an artificial neutral network to diagnose patients with *β*-thalassemia [6], and another study used a chatbot to diagnose users with various diseases and disorders [4]. None of the above studies used chatbots to assist with the care of *β*-thalassemia patients by providing the assessment information needed to keep track of their progress, which is the aim of this research. This study aims to develop a chatbot that can assist in the self-management of *β*-thalassemia by providing personalized, real-time feedback and in various tasks aimed at obtaining the assessment information required to monitor patients' statuses.

## 2. Methods

### 2.1. Framework Overview of Expert System Using Chatbot

**Figure 1:**
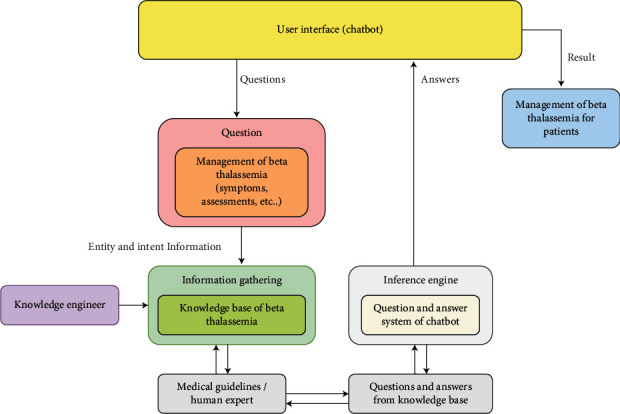
Framework of an expert system using the chatbot for the management of *β*-thalassemia patients.

The rule-based expert system, as shown in Figure 1, includes a *β*-thalassemia knowledge base, inference engine, knowledge engineer, and user interface. The knowledge base comprises three subphases: knowledge acquisition, knowledge representation, and knowledge verification. The inference engine simulated the interaction between the patients and the proposed chatbot and applied information about the management of *β*-thalassemia to identify the matching answers from inputs and collected knowledge in the knowledge base. A knowledge engineer is both an expert in knowledge engineering and a scientist who builds advanced logic on computer systems to simulate high-level, human cognitive decisions and tasks. The user interface was deployed to establish questions and present feedback. The framework used the waterfall model, whereby all requirements are collected in a knowledge base; the system design is represented in the form of questions and answers; and the implementation of the prototype is completed and sent to users for evaluation.

### 2.2. Rule-Based Chatbot

A chatbot is a computer program that is designed to communicate or converse with human users via the internet. Although most chatbots currently conduct simple dialogs, in which a customer asks one or more questions, certain domains require a more sustained dialog. The expertise contained in the knowledge base was utilized to address the problem of self-management in *β*-thalassemia and to generate recommendations on the required assessments and tests. The chatbot types are natural language processing (NLP)-based and rule-based. Typically, chatbots are rule-based, limited software systems with categories that automate human interactions [11]. Chatbots are easy to build and track in a certain predefined stream. Pre-set rules are established for communication. To receive an answer, the user input must conform to the predefined rules. Therefore, the rule-based chatbot is selected in this research. The rules are divided into two parts: If parts and Then parts. The If part is constructed in the questions and the user answers, while the Then part is automatically constructed as a recommendation from the collected knowledge.

#### 2.2.1. Knowledge acquisition

Knowledge about *β*-thalassemia management was gathered from documented and undocumented sources: human experts, books, and guidelines. First, a hematologist from King Abdulaziz University Hospital was interviewed about the general procedures performed to diagnose and treat *β*-thalassemia patients. Second, several medical books [12, 13] were selected to collect general information about the disease. Last, two guidelines were applied to gather information about the assessments and to monitor the patients: the clinical care of patients with thalassemia in Canada [14] and the management of transfusion-dependent thalassemia [15]. The knowledge gathered includes symptoms of *β*-thalassemia, categories of *β*-thalassemia, evaluation and required assessment, and abnormal test results, such as iron overload, low mean corpuscular volume (MCV), low mean corpuscular hemoglobin (MCH), and low hemoglobin (Hb) levels compared with normal test results.

#### 2.2.2. Knowledge Representation

The visual modeling approach is intended to enable the user to visualize and manipulate real-world issues using graphs. After the necessary information about *β*-thalassemia had been gathered, it was represented in the semantic network representation [16]. The figures present the knowledge represented as semantic network graphs using visual modeling techniques. These graphs are the final source of information needed to operate the question/answer system in the chatbot.

#### 2.2.3. Knowledge Verification

Two human experts examined the knowledge base and provided feedback. In response, some knowledge concerning *β*-thalassemia was corrected and developed in the correct form.

### 2.3. Development of the Chatbot

The collected knowledge was converted to a question/answer format to construct the chatbot. The chatbot-based expert system was built using Landbot [17], which uses approximately 50 products and services in technology, including the G2 Stack, HTML5, Google Analytics, and jQuery. Landbot uses website navigation tools with a familiar rich-text interface. First, possible questions and answers are listed in the knowledge base, as described in Table 1. The user should select from the dropdown list to answer a question. A feedback message occurs if the user writes his/her answer in the textbox. Second, the chatbot's semantic network is constructed in the Landbot workspace. Third, the knowledge engineer selected a suitable tool from the list to build the conversation: send a message, ask questions, follow operations, integration, power-ups, and bricks. Most of the chatbot questions were created using the “ask question” tool. Several components, including names, date, text, and buttons, are used. Last, the chatbot is published, and the initial draft can be customized using settings to provide greater flexibility in the interface design.

The chatbot was operated and targeted toward patients to manage their test results and to remind them of their *β*-thalassemia follow-up assessment in the form of messages. The chatbot provided questions with answer choices to be used by the patients. After the patients choose an answer, the chatbot comments based on their answer and continues the conversation.

### 2.4. Testing

Testing is a process used to evaluate the chatbot's functionality, with the intention of minimizing the risk that a problem will arise during its operation. Testing is also performed to determine whether the system meets the specified requirements. First, the chatbot should be easy to use, accessible, and understandable. Second, unambiguous instructions should be provided. Last, the chatbot should be designed to be compatible with any platform.

There are two major software-testing techniques: a static testing and dynamic testing [18]. Each of these methods is appropriate for detecting a certain form of error on the chatbot. First, a static testing is a technique that is used to check defects in software application without executing the code. Static testing is conducted to prevent mistakes at an early stage of development when they are easier to detect and correct. We performed a walkthrough for static testing. Second, dynamic testing is a form of software testing that takes place in an environment where the code is performed. This method of testing is used to verify the software's functional behavior and to ensure that it satisfies the user's needs and specifications. The validation process of software verification is known as dynamic checking. To check compliance with the user's needs and specifications, we created several questions in Arabic language, which are translated to into English, evaluate the chatbot with real patients. The patients will be asked to complete certain tasks and identify any problems or confusion they experience. During a typical evaluation, participants try to complete typical tasks while observers watch, listen, and take notes. The aim is to detect any usability issues and to assess participant satisfaction with the chatbot. A Google form was chosen to implement a survey distributed among *β*-thalassemia patients. The questions seek feedback on the patients' use of the chatbot as follows:

- Did you know about check-up times before you used the chatbot or did you learn about them from the chatbot?
- Did you know the required blood tests assessment time before you used the chatbot, or did you learn about it from the chatbot?
- Did you know the normal range of blood test results before you used the chatbot, or did you learn it from the chatbot?
- Is this chatbot easy to use?
- Does the chatbot make *β*-thalassemia easier to manage?
- Do you think managing *β*-thalassemia will be more efficient with this chatbot?
- Do you think easy access to chatbots will save you time and money compared with visiting health care providers?
- Do you prefer to visit health care providers from time to time to obtain information about the required tests during appointments or do you prefer using a chatbot at home to access this information?

## 3. Results

### 3.1. Testing results

Table 2 includes a live chat scenario to test the functional requirement of the chatbot.

To test user satisfaction with the user's needs and specifications, survey responses were received from 34 patients, of whom 72.7% agreed that the chatbot was user-friendly. This result indicates that most patients experienced no difficulty in communicating with the chatbot. Moreover, 82.4% of the patients agreed that the ease of use and accessibility of the chatbot could save them the time and money that they usually spend on hospital visits, 90.9% of the patients agreed that the chatbot made managing *β*-thalassemia easier, and 82.4% of the patients believed that the chatbot would enable more efficient management of *β*-thalassemia. All the above statements support this study's hypothesis: the chatbot is user-friendly, accessible, and affordable and makes managing *β*-thalassemia easier and more efficient. Furthermore, 26.5% of the patients reported having learned the timetable for their follow-up assessments from the chatbot, 20.6% of the patients reported having learned the timetable for their blood tests, and 24.2% of the patients learned their normal blood test results from the chatbot. Although these percentages are low, the network representations and tables of follow-up assessments and blood tests benefited several patients and may support them in the long term. Additionally, 32.4% of the patients prefer to access the chatbot at home rather than attending hospital appointments. Hospital visits can be uncomfortable for certain patients, and such patients may prefer accessing chatbots in their own homes.

### 3.2. Knowledge Base Representation

**Figure 2:**
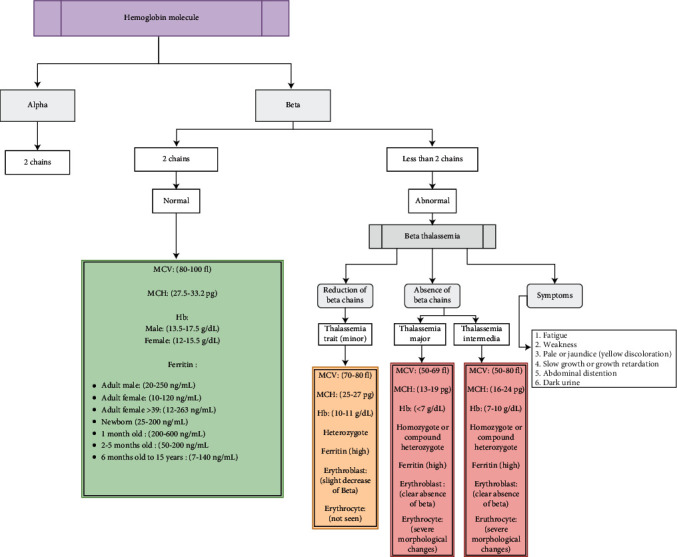
Semantic network representation of different stages of *β*-thalassemia symptoms and comparison between normal and abnormal results (MCV, MCH, Hb, Ferritin).

**Figure 3:**
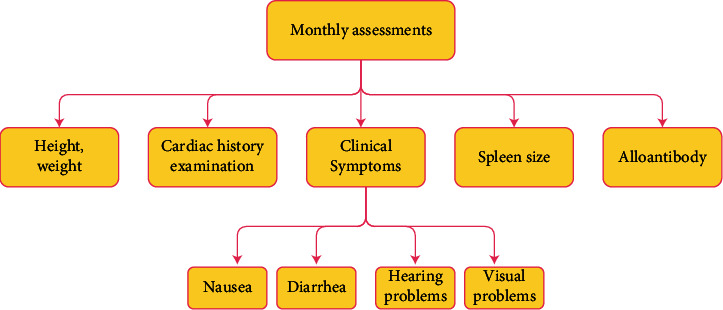
Semantic network representation of the monthly follow-up assessments.

**Figure 4:**
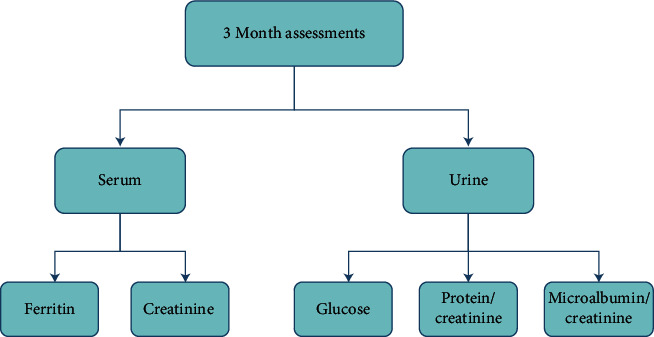
Semantic network representation of the three-month follow-up assessments.

**Figure 5:**
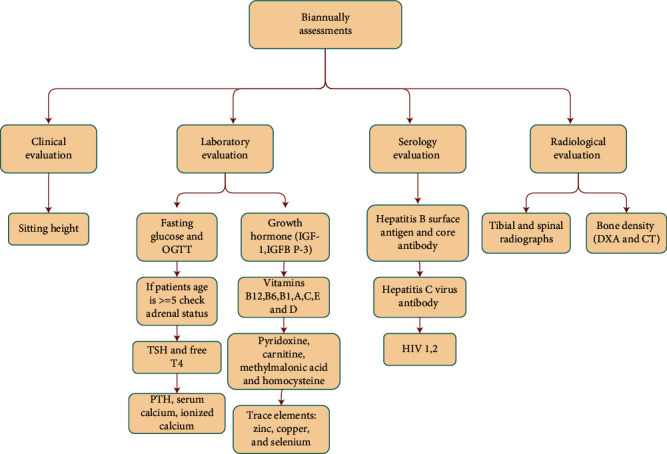
Semantic network representation of follow-up biannual assessments.

**Figure 6:**
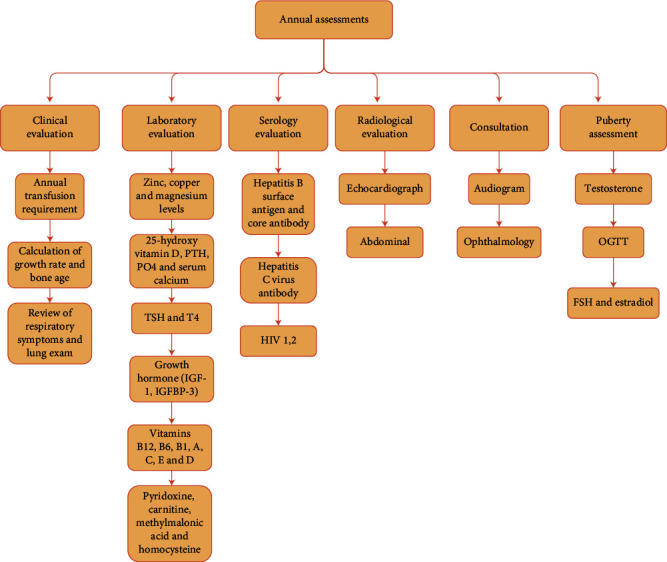
Semantic network representation of follow-up annual assessments.

*β*-thalassemia patients required several follow-up tests in monthly, three-monthly, biannual, and annual assessments. These assessments were represented in the semantic network representations, as shown in Figures 2–6, and were integrated in the chatbot to display personal feedback. The flowchart will be displayed based on a patient's query. For example, consider that the patient's answer in the chatbot indicates that he/she did not complete the Ferritin test. The chatbot will recommend performing the Ferritin test and display the flowchart that summarizes the Ferritin assessment due in months.

### 3.3. Prototype Implementation

**Figure 7:**
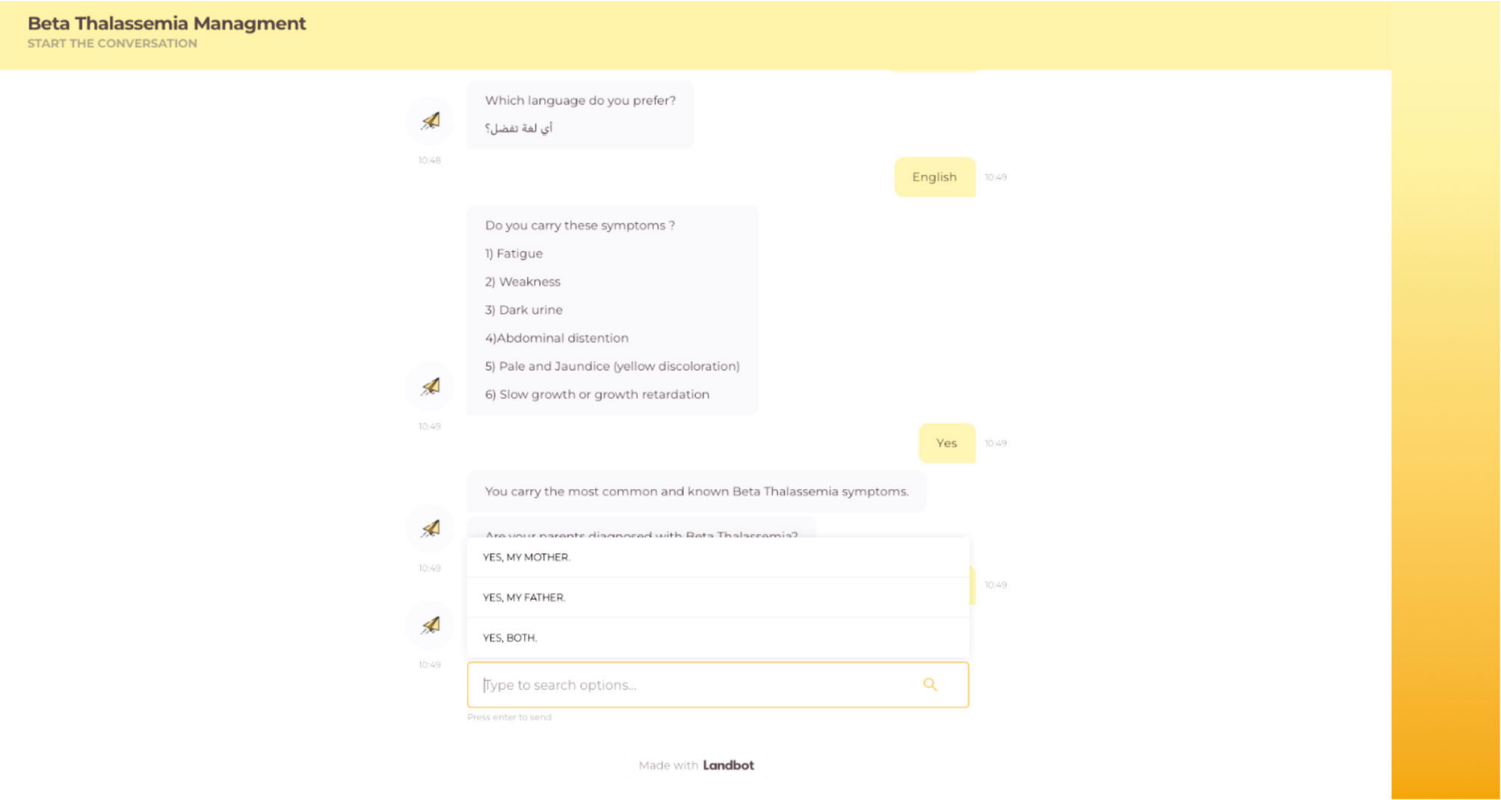
Screenshot of the conversation between the chatbot and user.

The chatbot-based expert system helps *β*-thalassemia patients understand what medical tests are necessary without face-to-face contact. The chatbot may be accessed via the following link: https://chats.landbot.io/v3/H-947072-772QZJR6XMJAGCJW/index.html. The link is to a site that shows a chatbot-based expert system for *β*-thalassemia that will answer patients' questions, perform personal assessment, and provide appropriate recommendations regarding the required tests. This information is provided in accordance with the guidelines used to construct the knowledge base.  Figure 7 shows a screenshot of the conversation between the chatbot and the user.

## 4. Discussion

The chatbot-based expert system for *β*-thalassemia management supports the perspective on health quality that aims to address patient safety and efficacy and to promote patient confidentiality and timeliness of care [19]. The chatbot recommends precise timing for required tests and assessments, which can assist in improving health outcomes and reduce the number of patient visits with health care providers for examination. The chatbots' efficacy was evaluated in accordance with medical practice guidelines. Patients may disclose more information to chatbots than to health care providers. The chatbots save time for both patients and health care providers by answering the most frequently asked questions. Patients can receive personalized recommendations without needing to visit their health care providers. Thus, a rule-based chatbot can help *β*-thalassemia patients better manage their own health by accessing appropriate information about the required tests and assessments. Although chatbots cannot effectively provide care according to the full extent of patients' needs, they can at least provide detailed clarification regarding patient assessments and recommend the required tests. This work can be integrated with the work published by Banjar et al. [7] to support *β*-thalassemia patients. The goal of this work was to develop a web-based expert system for *β*-thalassemia management that will provide treatment recommendations and support patients' long-term care.

## 5. Conclusion

In this study, a chatbot-based expert system was implemented to help *β*-thalassemia patients self-manage their health. The subphases involved in building a knowledge base comprise knowledge acquisition, knowledge representation, and verification. The chatbot design and medical knowledge were employed to implement the chatbot. The usability test highlighted the ease and benefits of using the chatbot. Most participants agreed that the chatbot could support them by providing the necessary information about their assessments and tests. We anticipate that chatbot technology has considerable potential to have a greater role in the medical field in the future. Our chatbot-based expert system will be expanded in a subsequent study to cover all medical complications associated with thalassemia, such as iron overload and splenomegaly, to provide the services that patients require.

## Figures and Tables

**Table 1:** Chatbot questions and answers knowledge base.

	Questions			Possible answers				
	1) Do you carry these symptoms (fatigue, weakness, pale and jaundice (yellow discoloration), dark urine , abdominal distention, slow growth or growth retardation)?	Yes	No					

Reply from chatbot		You carry the most common and known symptoms of Beta thalassemia.						

	2.1) Are your parents diagnosed with Beta thalassemia?	Yes, both	Yes, my mother	Yes, my father	No			

Reply from chatbot		If you obtain both of your parents mutant allies, you have Beta thalassemia major or intermedia. If you obtain only one, you have Beta thalassemia trait (minor).	2.2) Are your parents carriers?			Yes, both	Yes, my father	Yes, my mother

Reply from chatbot						If you obtain both of your parents mutant allies, you have Beta thalassemia major or intermedia. If you obtain only one, you obtain Beta thalassemia trait (minor).		

	3) Are your parents diagnosed with other hemoglobin disorders?	Yes, both	Yes, my mother	Yes, my father	No			

Reply from chatbot		If you obtain this allele, you have Beta thalassemia major or intermedia.						

	4.1) When was your last CBC blood test?	Less than 1 month	1 month or more					

Reply from chatbot			Retake test please					

			4.2) What is your gender?	Female	Male			

				(moves to female version of question 9)	(moves to male version of question 9)			

	5) What is your MCV level?	70-80 fl	61-69 fl	51-60 fl				

Reply from chatbot		Your MCV level is a bit low. MCV: 80-100 fl	Your MCV level is low. MCV: 80-100 fl	Your MCV level is very low. MCV: 80-100 fl				

	6) What is your MCH level?	25-27 pg	16-24 pg	13-15 pg				

Reply from chatbot		Your MCH level is a bit low. MCH: 27.5-33.2 pg	Your MCH level is low. MCH: 27.5-33.2 pg	Your MCH level is very low. MCH: 27.5-33.2 pg				

	7) What is your gender?	Female			Male			

	8) What is your Hb level?	Hb: 10-11 g/dL	Hb: 7-9 g/dL	Hb: <7 g/dl	Hb: 10-13 g/dL	Hb: 7-9 g/dL	Hb: <7 g/dl	

		Your Hb level is a bit low. Hb: 12-15.5 g/dL	Your Hb level is low. Hb: 12-15.5 g/dL. As thalassmia patient, your Hb level should be between 9 and 12	Your Hb level is very low Hb: 12-15.5 g/dL	Your Hb level is a bit low. Hb: 13.5-17.5 g/dL	Your Hb level is low. Hb: 13.5-17.5 g/dL	Your Hb level is very low Hb: 13.5-17.5 g/dL	

	9) Did you take a ferritin test?							

Answer 1	No							

Reply from chatbot	Moves to question 12							

Answer2	Yes							
Reply from chatbot	∗Provides table with tests∗ do you take your ferritin test accordingly to the table shown?							

	No							

Reply from chatbot	Moves to question 12							

	Yes							

	10) How old are you?	11) What is your ferritin level?			11) What is your ferritin level?			

	Newborn	>5000 ng/mL		510-5000 ng/mL		210-500 ng/mL		

Reply from chatbot		Your ferritin level is very high. Ferritin: 25-200 ng/mL		Your ferritin level is high. Ferritin; 25-200 ng/mL		Your ferritin level is a bit high. Ferritin: 25-200 ng/mL		

	1M	>5000 ng/mL		1000-5000 ng/mL		610-999 ng/mL		

Reply from chatbot		Your ferritin level is very high. Ferritin: 200-600 ng/mL		Your ferritin level is high. Ferritin: 200-600 ng/mL		Your ferritin level is a bit high. Ferritin: 200-600 ng/mL		

	2M-5M	>5000 ng/mL		510-5000 ng/mL		210-500 ng/mL		

Reply from chatbot		Your ferritin level is very high. Ferritin: 50-200 ng/mL		Your ferritin level is high. Ferritin: 50-200 ng/mL		Your ferritin level is a bit high. Ferritin: 50-200 ng/mL		

	6M-15Y	>5000 ng/mL		510-5000 ng/mL		150-500 ng/mL		

Reply from chatbot		Your ferritin level is very high. Ferritin: 7-140 ng/mL		Your ferritin level is high. Ferritin: 7-140 ng/mL		Your ferritin level is a bit high. Ferritin: 7-140 ng/mL		

	Adult	>5000 ng/mL	510-5000 ng/mL	130-500 ng/mL	>5000 ng/mL	510-5000 ng/mL	260-500 ng/mL	

Reply from chatbot		Your ferritin level is very high. Ferritin: 10-120 ng/mL	Your ferritin level is high. Ferritin: 10-120 ng/mL	Your ferritin level is a bit high. Ferritin: 10-120 ng/mL	Your ferritin level is very high. Ferritin: 20-250 ng/mL	Your ferritin level is high. Ferritin: 20-250 ng/mL	Your ferritin level is a bit high. Ferritin: 20-250 ng/mL	

	Adult >39 (this choice is included only for females)	>5000 ng/mL	510-5000 ng/mL	270-500 ng/mL				

Reply from chatbot		Your ferritin level is very high. Ferritin: 12-263 ng/mL	Your ferritin level is high. Ferritin: 12-263 ng/mL	Your ferritin level is a bit high. Ferritin: 12-263 ng/mL				

	12) What follow-up assessments would you like to see?	Annual	Biannually	3Month	Monthly			

Reply from chatbot		Chart of annual assessments	Chart of biannually assessments	Chart of 3 month assessments	Chart of monthly assessments			

	13) Would you like to see the other assessments?	Yes	No					
		Moves back to question 12	Glad to help you today, I'll chat with you another time!					

**Table 2:** Unit testing scenario for the chatbot.

Steps	User answers	Chatbot answers	The chatbot can respond to the user selection
The user clicks on the chatbot			Pass
Which language do you prefer?	English		Pass
Do you carry these symptoms? (1) Fatigue (2) Weakness (3) Dark urine (4) Abdominal distention (5) Pale and jaundice (yellow discoloration) (6) Slow growth or growth retardation	Yes	You carry the most common and known Beta thalassemia symptoms.	Pass
Are your parents diagnosed with Beta thalassemia?	Yes, my father.		Pass
Are your parents carriers of Beta thalassemia?	Yes, my father.	If you obtain both of your parents mutant alleles, you obtain thalassemia major or intermedia, if you only obtain one, you obtain thalassemia trait (minor)	Pass
Are your parents diagnosed with or carry other hemoglobin disorders?	Yes, my father.		Pass
When was your last CBC test?	Less than one month ago		Pass
What is your MCV level?	61-69 fl	Your MCV level is low. MCV: 80-100 fl	Pass
What is your MCH level?	20-24 pg	Your MCH level is low. MCH: 27.5-33.2 pg	Pass
What is your gender?	Female		Pass
What is your Hb level?	Hb: 7-9 g/dL	Your Hb level is low. Hb: 12-15.5 g/dL	Pass
Did you take a ferritin test?	No	Here are some information about ferritin concentration, LIC, and cardiac function tests. (attached file|)	Pass
What follow-up assessments would you like to see?	3 months	The recommended tests appeared	Pass

## Data Availability

The data that support the findings of this study are available on request from the corresponding author. The data are not publicly available due to privacy or ethical restrictions.
